# Metal Contamination Distribution Detection in High-Voltage Transmission Line Insulators by Laser-induced Breakdown Spectroscopy (LIBS)

**DOI:** 10.3390/s18082623

**Published:** 2018-08-10

**Authors:** Naixiao Wang, Xilin Wang, Ping Chen, Zhidong Jia, Liming Wang, Ronghui Huang, Qishen Lv

**Affiliations:** 1 Department of Electrical Engineering, Graduate School at Shenzhen, Tsinghua University, Shenzhen 518055, China; wnx17@mails.tsinghua.edu.cn (N.W.); cp17@mails.tsinghua.edu.cn (P.C.); jiazd@sz.tsinghua.edu.cn (Z.J.); wanglm@sz.tsinghua.edu.cn (L.W.); 2 Shenzhen Power Supply Co. Ltd., Shenzhen 518038, China; 13631561618@139.com (R.H.); lvqishen@sz.csg.cn (Q.L.)

**Keywords:** laser-induced breakdown spectroscopy (LIBS), metal contaminants, quantification, elemental distribution

## Abstract

The fast detection of classical contaminants and their distribution on high-voltage transmission line insulators is essential for ensuring the safe operation of the power grid. The analysis of existing insulator contamination has traditionally relied on taking samples during a power cut, taking the samples back to the lab and then testing them with elemental analysis equipment, especially for sugars, bird droppings, and heavy metal particulates, which cannot be analysed by the equivalent salt deposit density (ESDD) or non-soluble deposit density (NSDD) methods. In this study, a novel method called laser-induced breakdown spectroscopy (LIBS) offering the advantages of no sample preparation, being nearly nondestructive and having a fast speed was applied for the analysis of metal contamination. Several LIBS parameters (laser energy and delay time) were optimized to obtain better resolution of the spectral data. The limit of detection (LOD) of the observed elements was obtained using a calibration curve. Compared to calibration curves, multivariate analysis methods including principal component analysis (PCA), k-means and partial least squares regression (PLSR) showed their superiority in analyzing metal contamination in insulators. Then, the elemental distribution of natural pollution was predicted using LIBS to fully capture information about the bulk elements (Na, Ni, Cu, Mn, Ca, etc.) of entire areas with PLSR. The results showed that LIBS could be a promising method for accurate direct online quantification of metal contamination in insulators.

## 1. Introduction

Transmission line insulators are often contaminated with dust, bird droppings, soluble salts and metal particulates from Nature or surrounding factories. Therefore, pollution flashover more easily occurs, causing large-scale power cuts under wet conditions [1,2,3,4]. The contamination of insulators is profoundly complex, encompassing species such as NaCl, KNO_3_, NaNO_3_, SiO_2_, C, Al_2_O_3_, etc. In particular, insulators located near chemical plants, mines, etc. are more likely to attract electrically conductive metal particles, such as Cu, Mn, Ni, etc., which even in micro amounts may cause power frequency flashover accidents [5]. 

Serious partial discharge activity and flashovers on insulator strings occurred in 2010 and 2013 at the Ningxia Fuxiang 220 kV substation in China. Surrounding this were densely distributed thermal power plants, cement plants, chemical plants, etc. A variety of Na, Mg, Al, Fe, Mn, Zn, C, CaSO_4_ and semiconductors were adhered on the insulators, which distorted local electric fields by stacking them on a transformer sheathed coating, ultimately resulting in corona discharge [6]. 

Several researchers have explored the flashover performance of diverse types of contaminants on insulator surfaces. The flashover characteristics of sugar as a contaminant on glass insulators and RTV-coated insulators were worse than those of non-sugary contamination, and compared with non-RTV-coated insulators with non-sugary contamination, the flashover voltage of RTV-coated insulators covered with sugary contamination was higher [7]. The influence of salt mixtures on the DC flashover voltage discharge characteristics was studied in [8]. Mixing NaCl and other salts together as an artificial pollutant showed that different mixtures of salts had different flashover voltages and currents. The serious issue of flashover in outdoor insulators due to various environmental stresses and severe saline contaminant accumulation near shorelines was discussed in [9]. The model showed that the wetting and contamination deposition rate resulted in different surface discharge current characteristics of insulators in rain compared with those in cold fog, leading to different surface flashover voltages. Yang et al. [10] studied the electrical performance of aluminum phosphate as a contaminant that accumulated on the surface of insulators due to heavy use of aluminum phosphate fertilizer in farmland. However, these types of contamination needed to be collected and sent to the laboratory after an accident has occurred. Then, the contamination components are obtained by taking into account the surrounding environment and the test results, which made it difficult to detect the cause of accidents at the time, resulting in large-scale economic losses. Therefore, having an early warning of the possible evolution of a flashover incident via rapid and accurate detection of contaminant components and contents is particularly important.

At present, several methods, such as the equivalent salt deposit density (ESDD), non-soluble deposit density (NSSD), leakage current, and laser-induced fluorescence (LIF) methods are used to measure the level of actual contamination on insulators for decision making before flashovers occur [11,12,13,14,15]. However, the widely used ESDD and NSSD methods must be carried out after the samples are taken back to the lab and cannot effectively account for basic considerations, such as the solubility of components [16]. In addition, metallic particles cannot contribute to the ESDD because the insolubility still cause local flashovers. Additionally, the ESDD and NSDD methods cannot accurately reflect the species and concentration of pollutants. This makes it difficult to effectively judge the actual pollution level. The leakage current can be used to monitor the pollution levels of insulators online but can only predict the risk of a forthcoming flashover, not provide early warnings for operators [17]. In addition, the magnitude of the leakage current of the insulators would decrease with the decrease of the non-uniform pollution distribution in insulators [18]. Thus, the prediction accuracy of the leakage current and LIF methods remains to be studied. In addition, effectively characterizing the non-uniform distribution characteristics of contamination with these detection parameters is difficult. Therefore, a new method for the direct and accurate analysis of the types, concentration and distribution of pollution in insulators that could be performed on site without blackouts is of urgent need.

As a popular analytical method for qualitative and quantitative analysis of elements in materials, laser-induced breakdown spectroscopy (LIBS) allows rapid multi-elemental in situ sample analysis in different environments regardless of the presence of gas or water with a wide range of pressure and temperature regimes without sample preparation and has fast analysis speeds, online application, high determination sensitivity to ppm concentrations and non-contact non-destructive operation [19,20]. The NASA Mars Science Laboratory (MSL) launched the ‘Curiosity’ to Mars, which consisted of a LIBS spectrometer and a remote microimager (RMI) as part of ChemCam in late 2011 [21,22]. 

Many studies have been performed using LIBS. Rauschenbach et al. [20] studied the influence of the temperature, moisture and roughness of rocks with LIBS to maximize the sensibility and accuracy of results. Lanza et al. [23] studied weathered rocks in a Martian atmosphere using LIBS, scanning electron microscopy (SEM) and electron probe microanalysis (EPMA). Principal component analysis (PCA) was used to classify the profile data. Compared to SEM analyses, elemental information of larger areas of steel samples can be monitored due to the increased speed of LIBS measurements, while SEM analysis provides more precise morphological information, such as the shape and size [24]. The combination of energy dispersive X-ray fluorescence spectrometry (EDXRF) and LIBS was used to detect P, K, Ca, Mg, S, Fe, Cu, Mn and Zn in wheat flour [25]. LIBS coupled with multivariate analysis (PCA, PLSR and PCA-SVMR) was applied for the rapid detection of heavy metals in rice [26]. The methods to analyze rock or mineral composition could be used to analyze natural contaminants in insulators because the compositions mainly come from organic matter, dust, and mineral particles, which are similar to those of rocks or minerals. The composition of contaminants with mixed inorganic substances and organic matter causes photothermal ablation and photochemical ablation processes to occur simultaneously when the laser is applied, but the former process occurs mainly when the metal or rock is ablated, which makes little difference.

However, the application of LIBS in high voltage engineering is rare. Regarding the evaluation of aging states for external insulation composite materials in power systems, Wang et al. [27,28,29] studied the elemental compositions of newly prepared samples of room temperature-vulcanized and high temperature-vulcanized silicone rubbers using LIBS. C, O, Fe and Si were detected via LIBS. The results showed that LIBS did not affect the hydrophobicity of the ablated area and that the depth of the ablated craters had a linear relationship with the laser pulse number, which could be used to detect the aging layer depth and element distribution of the silicone rubber, as verified with the EDS results. 

With LIBS, metal contaminant samples consisting of conductive metal particles on the surface of insulators with an expanded elemental coverage were studied for the first time in this paper. The characteristic spectral lines were selected to analyze the element composition of artificial samples. Measurement parameters for LIBS were systematically optimized, while calibration curves and multivariate analyses were employed to calibrate the model for quantitative analysis. The elemental distribution of natural pollution in composite insulators was obtained using LIBS for the main elements of some areas, with the PLSR model applied for further interpretation. The use of LIBS to detect metal contaminant will give new improved methods for accurately diagnosing contamination components and content online to reduce the probability of flashover accidents. 

## 2. Materials and Methods

### 2.1. Experimental Setup

**Figure 1:**
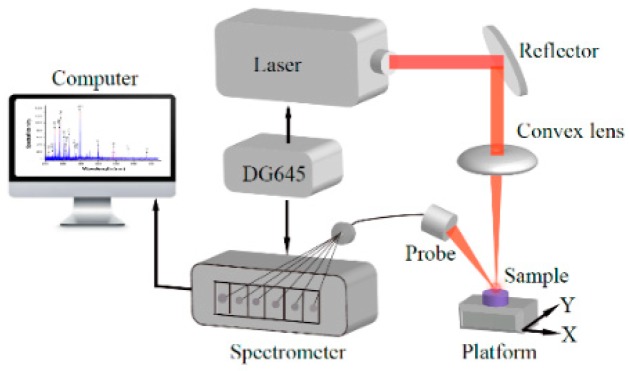
Schematic diagram of the LIBS experiment.

The experimental setup for the study is shown in Figure 1. The LIBS system consisted of a Q-switched laser (Beamtech Nimma-900, Beamtech China, Beijing, China), an Avantes optics spectrograph (Avantes Beijing, Beijing, China), including six CCDs and six spectrometers, and a Stanford Research System SRS DG645 Delay Generator (Stanford Research Systems, Inc., Sunnyvale, CA, USA). The laser, operating at a wavelength of 1624 nm, was focused on the surface of the samples for ablation using lenses and mirrors. The laser energy of the Nimma-900 system can be adjusted from 1 mJ to 900 mJ and the default of pulse width of the Nimma-900 (10 ns) was used. It was operated in the Q-switched mode at a repetition rate of 2 Hz. 

The diameter of the laser spot was 0.8 mm, and external gated detection was performed at a 3 μs gate delay and a 30 μs integration time. The gate delay was generated with a DG645, which then externally triggered the laser pulse. The experiment was performed in atmosphere. The surface elementals were vaporized, with a high temperature plasma formed by the laser. With a delay time after laser emission controlled by the DG645 and with control of the CCDs, the spectra were collected by an array of six plasma spectrometers for the different wavelength regions from 195 nm to 642 nm. Since the spectral line locations and intensities correspond to the types and concentration of the elements, respectively, the complex and variable information of the elements tested was acquired.

### 2.2. Sample Preparation

According to the IEC/TR2 61245:1993 standard [30], artificial contamination for the experiment was applied, in which the SDD and NSDD were selected based on the standard proposed pollution area division and the standard SDD value selection requirements. The pollution levels are divided into five grades according to the value range of SDD, that is “a”, “b”, “c”, “d”, “e” in IEC/TR2 61245:1993. The increase in SDD concentration from “a” to “e” indicates that the degree of contamination is more severe. The main parameters of the suspension glass insulator used in the transmission lines referenced are presented in Table 1. The average SDD values at both the top and bottom of the insulators ranged from approximately 0.02 mg/cm^2^ to 1.2 mg/cm^2^ (0.02, 0.05, 0.2 mg/cm^2^ represented three different kinds of pollution areas divided from “b” to “d” and 0.4, 0.8 and 1.2 mg/cm^2^ were all in the range of pollution areas of “e”). The NSDD value was always 2 mg/cm^2^. The kinds of mental contamination studied in this paper mainly corresponded to types of metal elements in actual contamination within the pollution area division of “e” in Ningxia, China and the concentration of these mental of the actual operating insulators in heavily polluted areas was obtained by SEM in advance to provide a reference for the types and concentration of the metal particles in the artificial contamination preparing. The content of the soluble salt was NaCl, and the ingredient of the NSDD was kaolin. The reference ranges of the special contaminant contents of naturally polluted insulator strings were obtained by measuring every insulator of two 110 kV insulator strings in different areas, such as in a copper mine, a nickel mine, a gypsum mine, and a manganese mine around Shizuishan City, China.

Four group configurations of standard artificial contamination with the addition of metal contaminants (Ni, Cu, Mn, and CaSO_4_) were named Group 1 to Group 4, as shown in Table 2. The NaCl and metal contaminants (Ni, Cu, Mn, and CaSO_4_) were purchased from Shanghai Chinese Medicine Reagent Group. The type and weight percent of each special contaminant are shown in the table, where the mass fraction percent of Ca was again calculated based on the ratio of the relative atomic mass of Ca to the molecular weight of CaSO_4_, similar to the procedure for Na. A total of four different metal concentrations containing NaCl and kaolin were used for the analysis where each group owned the same values of SDD represented by the mass fraction of Na. Additionally, all pellets were formed out of each sample using a pellet forming press under 10 tons of force, improving the ablation effect of the sample by LIBS.

### 2.3. Test Procedure

The samples above were placed on a motorized XY stage. The height of the electric stage was adjusted so that the optical fiber probe and the laser spot were refocused on the surface of the sample, and the XY position of the stage was adjusted to select the position of each test point. The following elements were measured: Na (589.592 nm and 588.995 nm), Ni (352.454 nm), Cu (216.509 nm and 217.894 nm), Mn (403.049 nm and 403.307 nm) and Ca (315.887 nm and 317.933 nm). The data were processed using peak integration. The influence of the LIBS system parameters, such as the delay time and laser energy, on the spectral results of the measured elements were optimized, and the RSD (relative standard deviation) was calculated first. The values of the delay time ranged from 0.5 μs to 9 μs, and the results were normalized with the value of each element at 0.5 μs on one sample. The laser energy ranged from 1 mJ to 520 mJ, with the test interval of 40 mJ. After obtaining suitable system parameters, all samples shown in Table 2 were tested. Five test points were randomly selected on the surface of each sample to reduce the effects of random error and the variation of the laser pulse energy on the experimental results. Each test point was continuously impacted 10 times at a frequency of 2 Hz via LIBS and averaged after normalization of each spectral result as the average test result of each point. The result of averaging the intensity of the corresponding LIBS spectra of these 5 points was taken as the average spectral intensity of the corresponding element of the sample.

**Figure 2:**
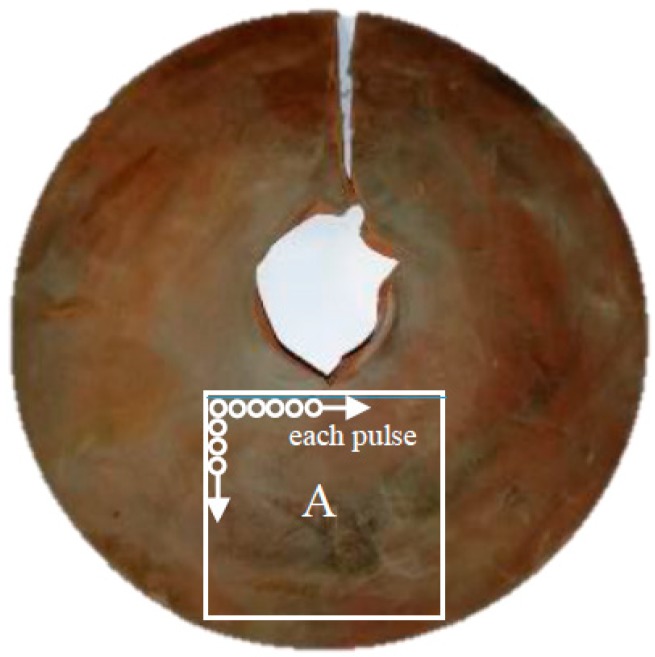
Surface conditions of the tested composite insulators.

The elemental distributions of the two-dimensional (2D) surfaces of the insulators were obtained to analyze the information on the types and locations of the contaminant. The contaminant was characterized by statistical analysis of the spectral wavelengths of the agglomerate elements. First, a piece of composite insulator with natural contaminant was selected, as shown in Figure 2. A piece was cut off labeled “A” in Figure 2 and placed on the electric X-Y platform. Then, the LIBS was performed on the selected area with the intensity of 20 × 20 arrays summing 400 points. The bombardment point interval was set to 80 μm with no overlap between different points. Each point was performed once with the energy of 80 mJ.

## 3. Results

### 3.1. System Parameter Optimization

To optimize the measurement process for multi-elemental analysis, several parameters need to be taken into consideration. In the experiment, the pulsed laser generates high-density energy that ablates and excites the materials in the surface of the sample. This creates a near-neutral, bright plasma composed of atoms, molecules and a large number of free electrons. At the beginning of the plasma cooling process, a strong continuous background spectrum was formed by the ionization of each element from bremsstrahlung and complex radiation, lasting hundreds of ns. Then, the electrons in the atoms and molecules in the excited state transition between different discrete bound energy levels, forming a linear spectrum that represents the characteristics of the atom, which is the atomic emission spectrum. The characteristic wavelength and corresponding intensity of the spectral lines are associated with the type and content of the elements measured, which is the basis for the qualitative or quantitative analysis of elements lasting several μs [31,32]. 

**Figure 3:**
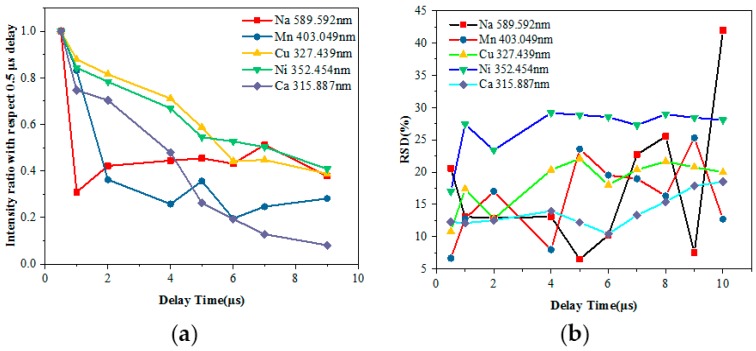
The influence of the delay time on the measured elements: (**a**) Normalized ratio with respect 0.5 μs delay time curves; (**b**) RSD curves.

Thus, the LIBS signal for the analyzed main components of the sample (Na, Ni, Cu, Ca and Mn) should be optimized to obtain the highest and most stable signal rather than one affected by the self-absorption. To ensure complete ablation of the sample material across the entire area covered by the laser beam, the gate delays between the laser output and spectrometer were taken into consideration for the optimization. The ratio of each point was calculated using normalization, which divided the spectral intensity of each delay time by the intensity of the delay time at 0.5 μs. The relative standard deviation (RSD) reflecting the precision of the measurement was considered. Figure 3 shows the influence of the delay time on the spectral intensity and the resulting RSD of different elements measured using LIBS. The RSD value can be expressed as in Equation (1):

(1) $$\left\{ \begin{array}{l} RSD=\frac{\sqrt{\frac{\sum_{i=1}^{n}{\left(x_{i}-\bar{x}\right)}^{2}}{n-1}}}{\bar{x}}\times 100\%\\ \bar{x}=\frac{x_{1}+x_{2}+\cdots +x_{n}}{n} \end{array}\right.,$$

where *x*_1_, *x*_2_, and *x_n_* are repeated measurements, $\bar{x}$ is the average of the repeated results, and *RSD* is the relative standard deviation.

There are several characteristic spectral lines for each element, which show nearly the same trend with increasing delay time. Therefore, we selected just one line for each element. When the delay time was 0.5 μs, the continuous background spectrum process was not yet complete; as the time delay increased, the spectral intensity of each element significantly reduced and the RSD of the measured elements slowly increased before gradually stabilizing, except for Na. When the delay time increased from 1 μs to 9 μs, the ratio of Na fluctuated within 0.3 to 0.45, and the RSD values of Na were volatile since it is an alkali metal element and easily ionizable. In addition, the atomic emission spectra occur within 1 μs, which causes the ionization number of Na received by the system to decrease randomly, reducing the accuracy of the measurement as the delay time increases. 

Considering the line intensity and RSD with the delay time, the normalized ratio above 0.4 and RSD lower than 20% were selected, as the delay time was optimized from 2 μs to 4 μs. The 3 μs gate delay was used in a later experiment. If a particular element is needed for analysis, a range of delay times with normalized ratios above 0.5 and a minimum of RSD can be selected. 

**Figure 4:**
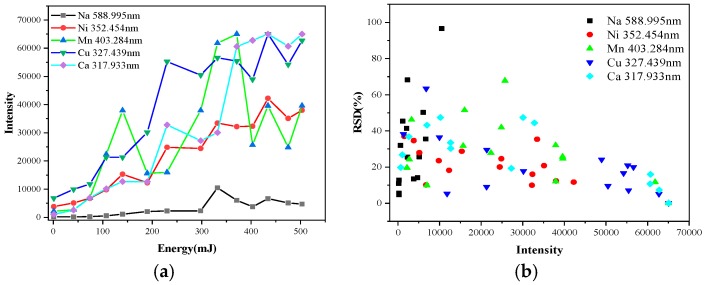
The influence of the pulse laser energy on the measured elements: (**a**) Intensity curves; (**b**) RSD distribution.

The influence of the laser energy on the spectral intensity and RSD of different elements measured using LIBS is shown in Figure 4. The intensity of the spectral lines increased as the laser energy per pulse increased. The intensity of Mn fluctuated greatly because the particle size of Mn was greater than 250 μm at 50% distribution, while those of Ni, NaCl, Cu, and CaSO_4_ were between 31.35 μm and 48.385 μm by Laser Particle Size Analyzer (Mastersizer 2000, Malvern Panalytical, Malvern, UK). This leads to an uneven distribution of Mn compared with the other special contaminants of the same nature, causing insufficient material ablation while testing different points. The RSD is related to the sample concentration and spectral line intensity and is influenced by the spectral analysis conditions and instrument performance. From the RSD distribution in Figure 4b, with the increase of the intensity, the RSD of almost all elements descended slowly, which indicated that increasing the energy can effectively improve the accuracy of the results. However, the energy of 110 mJ could only remove contamination that was within hundreds μm of the surface of the insulators in our experiment at once. Then, laser energy per pulse beyond 110 mJ may damage the surface of the insulator. Furthermore, increasing the laser energy gave some elements enough excitation energy to cause intensity saturation or self-absorption, which would have produced a negative effect. Thus, the laser energy was adjusted to 80 mJ in this study.

### 3.2. Calibration Curve Method

**Figure 5:**
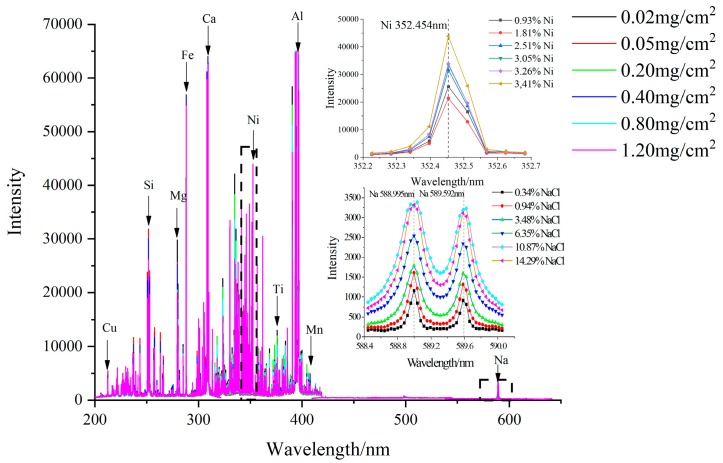
Spectral intensity distribution of different sampling points of Group 1.

The average spectral lines of the studied Cu, Ca, Ni, Na and Mn intensity of six different artificial contaminants from Group 1 were illustrated in Figure 5. Also, NaCl and Ni as key values in Group 1 are illustrated in the upper left and lower left corner of Figure 5. The figure showed that the intensity of the spectral lines would vary with changes in the elemental weight percent. Most of the emission lines were clustered below 400 nm, and increasing the Na and Ni concentration could lead to an increase in the Na emission at 589.592 nm and 588.995 nm and the Ni wavelength at 352.454 nm. According to the NIST database [33], other main spectral emission lines that correspond to Cu (216.509 nm and 217.894 nm), Mn (403.049 nm and 403.307 nm) and Ca (315.887 nm and 317.933 nm) were also acquired. These lines are generally resonance lines of the elements, and there was no overlap among them.

The average LIBS spectra of the samples, including 50 shots for each sample (five locations and ten excitations per location), were averaged to weaken the influence of the matrix effect. The peak intensities of the elemental emission lines were calculated by averaging the values based on a normal distribution. Then, the spectral data in different wavelength ranges were preprocessed by normalization to effectively improve the influence of various non-target factors on the spectral signal (energy hunting, resolution difference, exotic environment, etc.). This was done to improve the sensitivity and separation rate of the data to enhance the prediction ability and robustness of the model. The normalization method used was area normalization, which divided the spectrum collected from the spectrometer by the sum of the area of the entire spectral data and considered the difference of baselines for different CCDs. Each channel was normalized separately.

**Figure 6:**
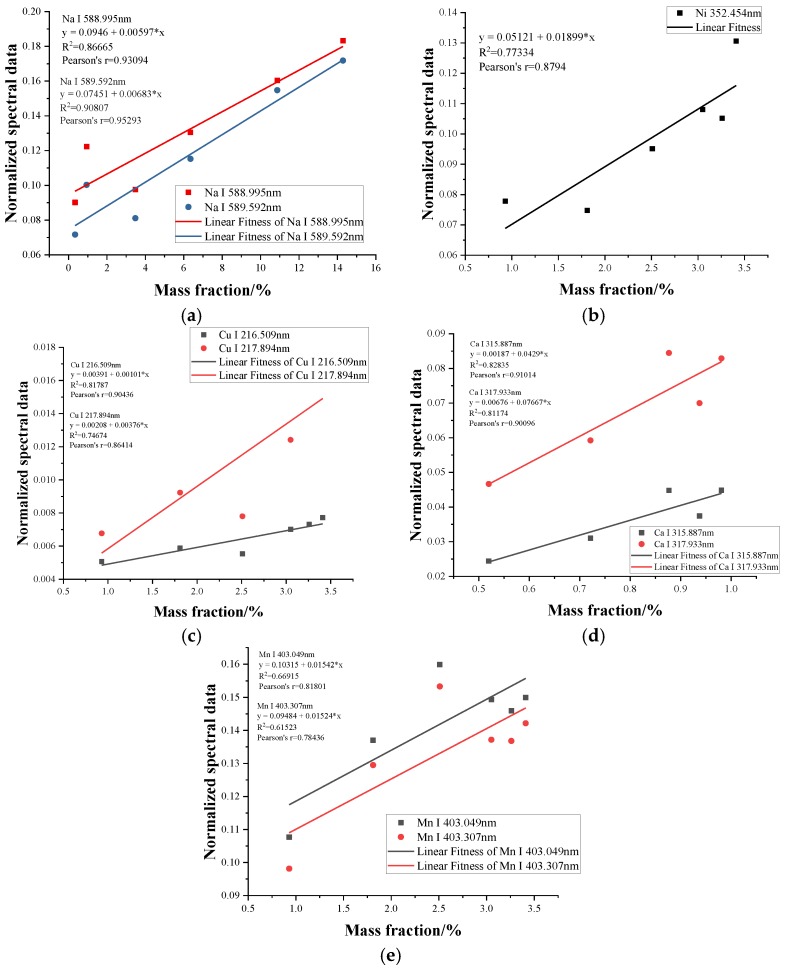
Linear relationship between the ion concentration of elements and their corresponding normalization spectral data, where (**a–e**) correspond to Na, Ni, Cu, Ca and Mn.

The calibration curve method was obtained with correlation coefficient (R^2^) values of 0.86665 and 0.90807 for Na (589.592 nm and 588.995 nm), 0.77334 for Ni, 0.81787 and 0.74674 for Cu (216.509 nm and 217.894 nm), 0.82835 and 0.81174 for Ca (315.887 nm and 317.933 nm) and 0.66915 and 0.61523 for Mn (403.049 nm and 403.307 nm), as shown in Figure 6. The quantitative analysis of the special contaminants can be done if we have sufficient samples and can find a database of different samples. It is worth noting that the slopes of the calibration curves corresponding to different wavelengths of the same elements, such as Cu, were quite different, which may cause errors in the calibration results when the single variable wavelength is used to characterize the calibration curves.

To evaluate the measurement sensitivity and precision, the limit of detection (LOD) was calculated, as in Equation (2):

(2) $$LOD=3\times \frac{S.D.}{S},$$

where *S.D.* is the standard deviation of the response, and *S* is the slope of the calibration curve.

The smallest LOD was selected in the calculation results at different wavelengths of the same element. From the known primary slope and background intensity, the remaining surface contamination could be estimated to be in the ppm range, as shown in Table 3. Particularly, Na (589.592 nm), Ni (352.454 nm), Cu (324.754 nm), Mn (403.049 nm), and Ca (317.933 nm) emissions could be maintained beyond 382.03 ppm, 65.071 ppm, 41.191 ppm, 115.073 ppm and 16.316 ppm. Generally, Ca exists in pollution in the form of compounds, such as CaCO_3_ and CaSO_4_; assuming that the LOD of Ca in those compounds was 16.316 ppm, the LOD of CaCO_3_ and CaSO_4_ would be 55.4744 ppm and 40.79 ppm, respectively. In some studies, researchers obtained lower detection limits, as low as 69 ppm for Na [34] and even 0.1 ppb via dual-pulse and crossed-beam lasers for Na on a water film [35]. However, assuming that the LOD of Na in NaCl was 382.03 ppm, the weight percent of Na would be 0.0382%, which is within the pollution area division of b, as Table 2 shows, and is adequate for detecting the contaminant on the surface of insulators in moderately or severely polluted areas. The LOD of other metallic elements was satisfactory for the detection of elements on insulators near factories and mines or near urban roads. In [36], Gondal studied the LOD of Na, Ca, Mn, etc. in cable via LIBS. They obtained lower LOD of Na (589.59 nm) at 39.56 ppm and Mn (257.61 nm) at 4.2 ppm while obtained higher LOD of Ca (393.36 nm) at 43.2 ppm. The reason for the difference of Ca, Mn might be that the metal ions detected in this paper are all simple materials, while the components in [36] are compounds. Na, a kind of alkali metal, was greatly influenced by environment and instrument parameters, which resulted in great difference of experimental results. In addition, it was worth noting that the LOD results were different at different wavelengths of the same element.

### 3.3. Multivariate Methods for Classification and Predication

The types of contaminants in complex samples would interfere with each other, and the analysis of a single element would not be able to provide the relationship between the contents and intensity because the relationship between the LIBS intensity and elemental content is typically complicated by matrix effects, such as laser-to-sample coupling efficiency, self-absorption and trace element abundances [37]. Quantitative LIBS analysis on special contaminants would present a challenge because of the widely varying elemental compositions. Encouragingly, multivariate methods that use information from the entire spectrum or some observed emission lines rather than a single emission line have been shown to yield more precise results. In this paper, the K-means, PCA and PLSR were employed for qualitative or quantitative analysis as a means of exploiting the matrix effects. 

**Figure 7:**
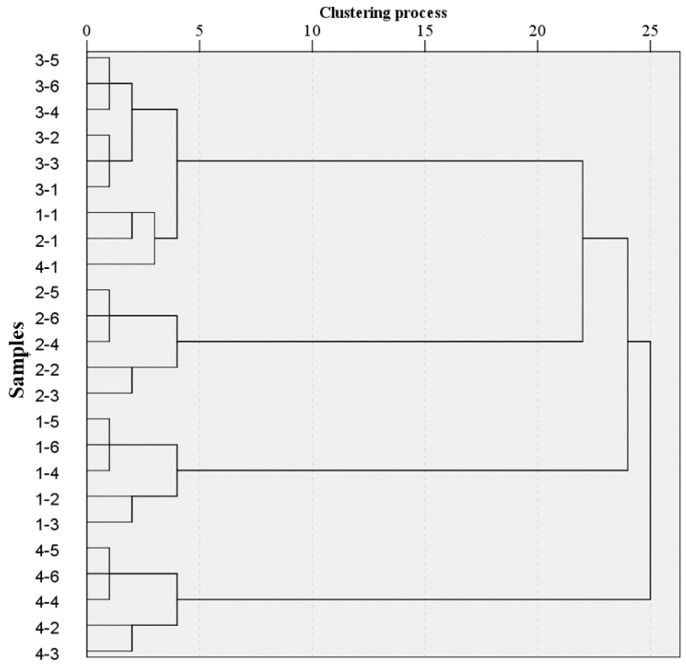
Clustering tree obtained using K-means.

K-means, an unsupervised method, works iteratively to group samples by means that reveals their similarity with measured variables [38]. Classification based on observed emissions can obtain acceptable results. According to Table 2, the total number of test samples of 24 and the k number of 4 were set previously. At the beginning of the algorithm, four samples were randomly selected as the initial mean vector, then the Squared Euclidean Distances between other samples and the four initial vectors respectively was calculated. Compared with the calculated four distances of each sample, the shortest distance was chosen and assigned to the clustering group. For example, compared with the four distances (A1, A2, A3, A4) of sample 4-6 which correspond to four vectors (1-3, 2-3, 3-3, 4-3), the shortest distance is A4. Then, the sample 4-6 was clustered to 4-3. Similarly, the current clustering groups could be obtained by calculating the above process for all the samples of the dataset after the first iteration. Repeat the iterative procedures above until the clustering groups didn’t change. Seeing to the left from the right side of the graph in Figure 7, two clustering groups were formed provisionally after the first iteration, three clustering grouped together after the second iteration and finally iterative procedures ended until four clustering groups were invariant. In Figure 7, the classification accuracy was 87.5%, where 1-1, 2-1 and 4-1 and all samples of Group 3 (3-1 to 3-6) clustered together, and other categories besides X-1 of each were classified as shown in Figure 7.

**Figure 8:**
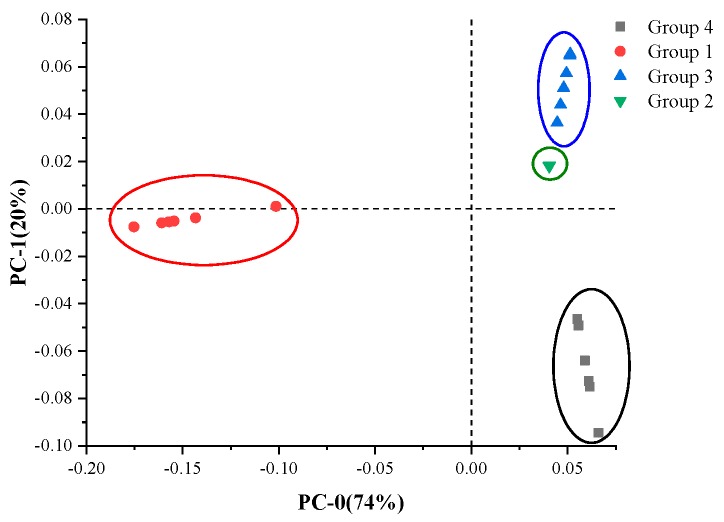
Score plots of PC-0 and PC-1 obtained using PCA.

The PCA classification method was used to obtain a more accurate classification result. The pattern recognition method includes both supervised and unsupervised approaches. K-means belongs to the former, while PCA belongs to the latter. Figure 8 is a two-dimensional scatter plot of scores for two specified components (PC-0 and PC-1) from PCA, where 94% of the variation in the data was described by the two components. Four distinct species of special contaminants were clearly classified, as shown in Figure 8. Group 1, Group 3 and Group 4 were clearly separated, where the samples from each category had slight differences, leading to a small separation in each cluster, while the cluster of Group 2 gathered at almost one point, indicating that the Group 2 samples had extremely strong similarities in this analysis.

Furthermore, multivariate metrology analysis can make full use of the spectral information to establish a model between contamination content and spectral intensity to perform a quantitative analysis. PLSR was applied in the prediction of the content in metal contamination. PLSR models both the X-matrices and Y-matrices simultaneously to find the latent variables in X to predict the latent variables best in Y. In the model input tab, the matrix in which the normalized intensities of Na, Ni, Cu, Mn and Ca were set as X-matrices and the weight percentages of the observed elements above were set as Y-matrices was analyzed in the data frame where all the weights of the variables were 1 and the Kernel PLS was selected for the model calibration. PLSR uses a full cross-validation in which one can choose the number of segments of a data set, and then, the cross-validation procedure randomly selects the number of samples to take as an unknown dependent variable. This provides a good way to check the quality of the regression model. If the model gives generally good results, the plot will show points close to a straight line through the origin with slope close to 1 (y = x), with R^2^ close to 1. The four categories (Group 1 to Group 4) were used as the training and cross-validation data sets for the model. 

**Figure 9:**
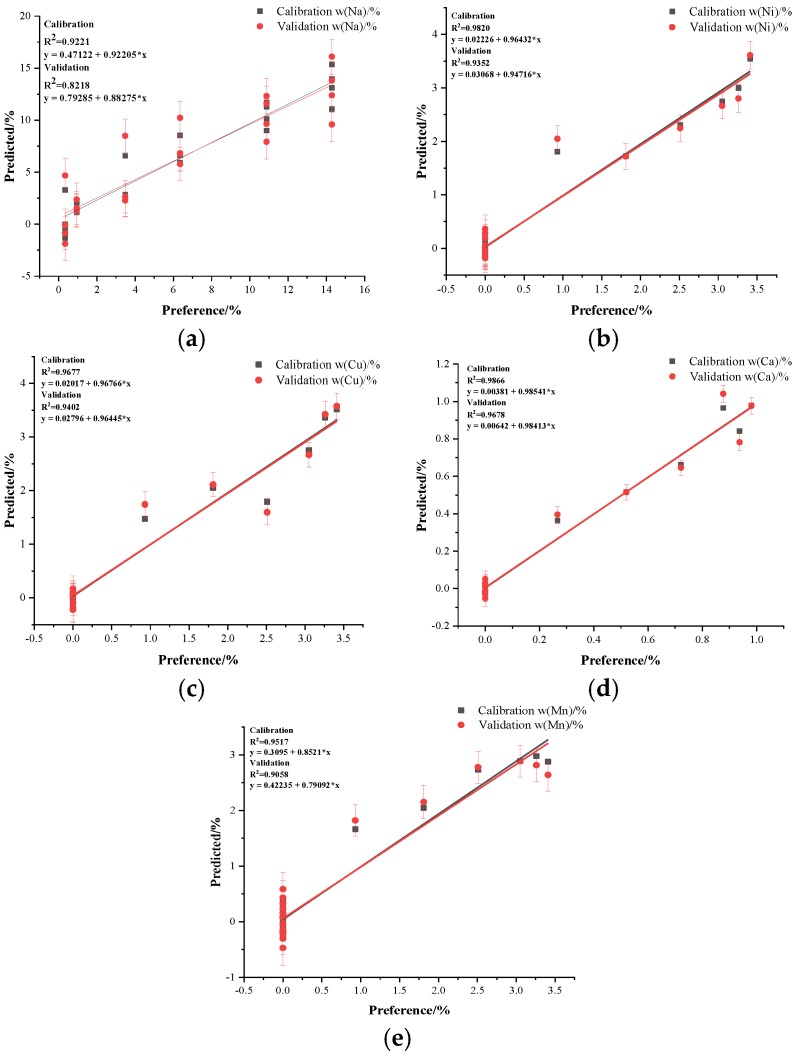
Model and cross-validation plots produced with the PLSR, where (**a**–**e**) correspond to Na, Ni, Cu, Ca and Mn.

Figure 9 contains the calibration and validation plots for all five elements analyzed. These plots demonstrated highly consistent results regardless of the calibration or validation set curves. The PLSR model based on the observed emissions obtained acceptable results. The R^2^ values for Na, Ni, Cu, Ca and Mn were greater than 0.8, and the fitting lines were nearly through the origin. The RMSEV (root mean square error of cross validation) is a measure of the dispersion of the validation samples around the regression line when cross-validation is used. 

The closer the RMSEV values of the calibration and validation are, the better the data are modeled. The results of the RMSEV for the calibration and validation of the observed elements were typically close, as shown in Table 4 for Ni, Mn, Cu and Ca. Additionally, the average values of standard error (SD) for validating Na, Ni, Cu, Ca and Mn in the model were 1.620, 0.250, 0.238, 0.043 and 0.291 which indicated that the results predicted by the PLSR model can be well distributed around the mean value. Also, the regression coefficient in the PLSR could be used to calculate the values, which was convenient for offline numerical predictions. 

Overall, some studies show that the PLSR was generally the most accurate method for the analysis of typical geologic materials [39,40]. The experimental results in this paper verified the above conclusions. Based on this model, a database possessing the types and contents of observed elemental information can be established, through which the types and concentration contents of the contamination can be predicted, even when the spectral data are collected using only a LIBS analysis on insulators onsite in the future.

### 3.4. Elemental Mapping of a Composite Insulator with Natural Pollution

The distribution of contamination in operating insulators is usually uneven due to the multiple effects of wind, rain, fog, etc., and the non-uniform pollution distribution in insulators has a significant effect on the flashover characteristics. Thus, it is necessary to acquire the distribution and concentration of contaminants in insulators. In this paper, types and contents of the observed elements of some areas were obtained using LIBS, and the elemental mappings of the contamination was performed. The measurement parameters determined during the optimization procedure described above were used for the elemental mapping experiment. 

**Figure 10:**
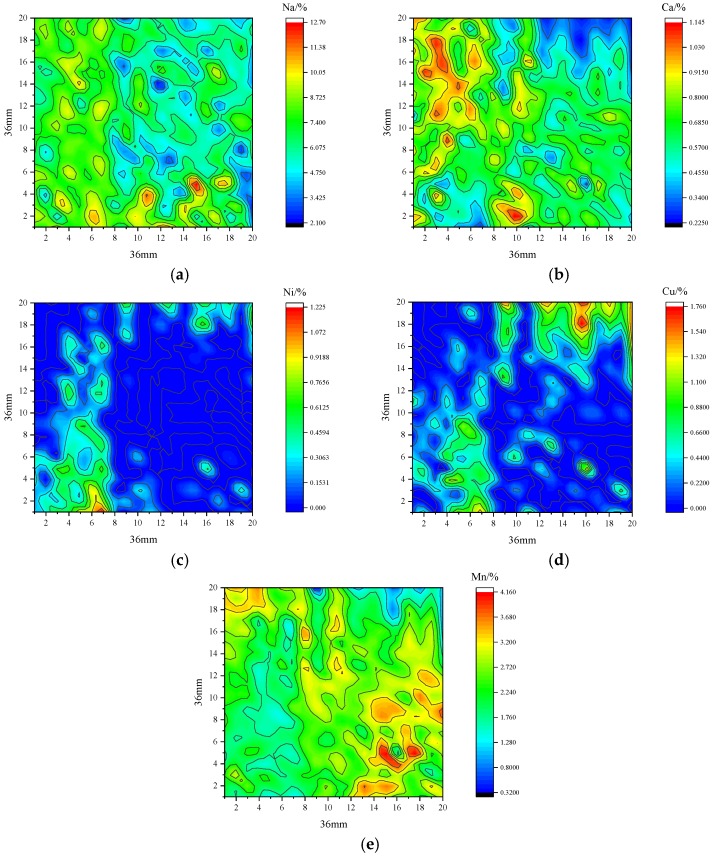
Elemental mapping produced with the model using PLSR, where (**a**–**e**) are Na, Ca, Ni, Cu and Mn.

After the intensity of 20 × 20 arrays was obtained with LIBS by summing 400 points in a region of 36 × 36 mm, the content was predicted with the PLSR model above after normalization. The 2D elemental distributions on the surface of the insulator could be obtained to analyze the type and location of the contamination agglomerates, as shown in Figure 10, where the envelope lines in the graphs are the lines of equal concentration. The results were instructive for the quantitative analysis of contamination using LIBS. 

First, the distribution of the observed elements on the sampled surface were different, and the distributions of the same element on the sample surface were also uneven. This may lead to inaccurate measurements of the contamination composition in one sample when only a few points on the surface of the sample are normally sampled. Additionally, Na and Ca, as the main components of natural pollution on insulators, were distributed similarly over the whole area, and the average concentration of Na at 6.8 ± 3.7% was much stronger than that of Ca at 0.65 ± 0.39%. Ni and Cu are only distributed in marginal areas, which can suggest that some Ni and Cu metal particles might attach to the surface. During operation, the clusters of Ni and Cu could distort local electric fields by stacking them on the insulators, which would produce corona discharge or brush discharge to accelerate aging of the insulators. Additionally, the distribution of Mn on the sampling surface was uniform, with the average weight percent of 2.4 ± 1.2%, relatively higher than those of Ni and Cu. There was an obvious accumulation phenomenon in the distribution of metal particles, which might be a homogeneous accumulation of Mn compounds on the sampling surface.

Overall, elemental mapping provided a new method to analyze the distribution of elements online, which can represent the accumulation of elements over the entire surface. Compared to the SEM analysis, elemental information of larger areas of materials of several cm^2^ could be monitored due to the speed of LIBS measurement, while SEM analysis provides more precise information, such as shape and size, within an area of some μm^2^. More precise methods of determining element content by LIBS need further study.

## 4. Conclusions

Metal contaminants of Ni, Cu, Mn, and CaSO_4_ in insulators in high-voltage power lines were detected using LIBS. LIBS can analyze samples without sample preparation and possesses high determination sensitivity to even ppm concentrations in a non-destructive way. For the adjustment of contamination components and content of insulators online, LIBS has the potential to detect the metal contaminants on insulators, which would be meaningful for further study. In this study, measurement parameters were optimized with respect to the spectral intensity and RSD of measured elements. Calibration curves were obtained between the concentration and intensity, and the LOD of the observed elements was calculated. K-means, PCA and PLSR were employed to analyze the LIBS results as a means of exploiting the matrix effects. PCA had better classification results than K-means, and PLSR was applied to predict the chemical compositions of special contamination. The PLSR model fit the training data perfectly. The distribution and accumulation of the intensity of different elements in a region of 36 × 36 mm were obtained using LIBS scanning. After accumulating the PLSR model, the elemental mapping of the content of the observed elements was obtained for further interpretation, which may be of use in understanding the entire distribution of insulator contaminants. LIBS is thus an ideal tool to characterize the distributions of contaminants on insulators.

## Figures and Tables

**Table 1:** Main parameters of the suspension glass insulator referenced.

Type	Top Surface (cm^2^)	Bottom Surface (cm^2^)	All Surfaces (cm^2^)	Creepage Distance (cm)	Diameter (mm)
XP-70	674	917	1591	280–300	255

**Table 2:** Type and mass fraction of the metal contaminants.

	Type	Content					
Group1	Ni (%)	0.93	1.81	2.51	3.05	3.26	3.41
Group2	Cu (%)	0.93	1.81	2.51	3.05	3.26	3.41
Group3	Mn (%)	0.93	1.81	2.51	3.05	3.26	3.41
Group4	Ca (%)	0.26	0.50	0.67	0.80	0.85	0.88
Each Group	Na (%)	0.34	0.94	3.48	6.35	10.87	14.29

**Table 3:** LOD values for measured elements.

Type	Na	Ni	Cu	Mn	Ca
**LOD (ppm)**	382.03	65.071	41.191	115.073	16.316

**Table 4:** The RMSEV of the PLSR model.

Type	Na	Ni	Cu	Ca	Mn
**Calibration**	1.43	0.22	0.21	0.04	0.26
**Validation**	2.16	0.29	0.29	0.06	0.36
